# The landscape of medical care consumption in Israel: a nationwide population cross-sectional study

**DOI:** 10.1186/s13584-022-00542-9

**Published:** 2022-11-10

**Authors:** Yoni Yosef, Alexander Kiderman, David Chinitz, Amnon Lahad

**Affiliations:** 1 Israel Defense Forces, Medical Corps, Jerusalem, Israel; 2 Clalit Health Services, Jerusalem, Israel; 3 School of Public Health, The Hebrew University of Jerusalem, Jerusalem, Israel

**Keywords:** Israel, Universal health care, Ecology, Primary health care, Consumption, Illness perception, Health perception

## Abstract

**Background:**

The Ecology of medical care was first published in 1961. The graphical square model showed that 75% of the population in the US and England experience a feeling of illness during a given month, 25% seek medical help and only one percent are hospitalized. In 2001, Green and colleagues found the same findings despite the many changes that occurred over the past decades. The frequency of illness, the desire for assistance and the frequency of seeking and getting medical assistance may differ in different populations due to cultural, economic, social, demographic background and local Health policy. This work describes the ecology of medical care consumption in Israel for the first time and examines the socio-demographic effects on consumption.

**Methods:**

This is a Nationwide cross-sectional study. A telephone survey was conducted among a representative sample of the adult population (> 15 years) in Israel. Subjective morbidity rate in the preceding month, the rate of those considering medical assistance and those who got assistance were calculated. Correlation between socio-demographic variables and patterns of morbidity and medical care consumption was examined using a t-test and chi square for continuous quantitative and categorical variables. Logistic regression was used for multivariate analysis.

**Results:**

A total of 1862 people participated; 49.5% reported having symptoms in the previous month, 45% considered seeking medical advice, 35.2% sought out medical assistance and only 1.5% were hospitalized. The vast majority chose to contact their family physician (58%) and the primary care setting provided their needs in 80% of the cases; Subjective morbidity and medical care consumption differed significantly between Israeli Jews and Arabs. Gaps in the availability of medical services were observed as residents of the periphery forewent medical services significantly more than others (OR = 1.42, *p* = 0.026).

**Conclusions:**

Subjective morbidity is less common in Israel than in other countries, but paradoxically consumption of medical services is higher. An Israeli who feels ill will usually consider receiving assistance and will indeed receive assistance in most cases. However, a greater tendency to forego medical services in the periphery indicates barriers and inequality in the provision of health services. Different cultural perceptions, lack of knowledge and low accessibility to medical services in the periphery probably contribute to the contrast shown between low consumption of medical services and high prevalence of chronic illness in Arab society. The prevailing preference for family medicine and its ability to deal with most requests for assistance suggest that strengthening family medicine in the periphery may reduce those barriers and inequalities.

**Supplementary Information:**

The online version contains supplementary material available at 10.1186/s13584-022-00542-9.

## Introduction

The ecology of healthcare consumption was first described almost 60 years ago by White and colleagues [1]. They conducted a telephone survey among a sample of U.S. and England residents and found that a large proportion of the population (about 75%) experienced illness during a given month, 25% sought out medical care and less than one percent were hospitalized. The results of the study were presented graphically in a square diagram. In 2001, Green and colleagues repeated this study in the United States [2] and found similar findings, despite the decades that had passed and the monumental changes that had occurred in the world of medicine. The study was then repeated in Europe and Asia, with similar results [3–5].

Israel has unique characteristics that may influence the "evolution" of consumption, including a highly diverse and large immigrant population and universal health care [6], two variables known to influence health care consumption [7–10]. National efforts in community-based preventive medicine, health promotion and education, as well as an increase in the overall standard of living and socioeconomic status of the population, may also affect not only illness prevalence but also healthcare consumption and management [11]. There are also marked differences between the geographic center and peripheral areas, affecting overall health status and access to medical services [12].

Despite these unique variables, no ecological review describing the landscape of the Israeli healthcare system has ever been conducted. Surveys of the Central Bureau of Statistics and HMO data describe the actual consumption of medical services, without examining the prevalence of self-assessed morbidity and the demand for medical services. Descriptions of consumption of services do not enable identification of populations that experience morbidity but nevertheless forego medical services and does not indicate how socio-demographic characteristics affect perceived morbidity and actual utilization of health care. We will describe, for the first time, the ecology of the consumption of medical services in Israel and compare different strata within the Israeli population.

## Methods

Study Design: A nationally representative cross-sectional study of permanent Israeli residents. Using a landline telephone database, a computerized randomized household sampling was conducted each month, between August 2015 and July 2016. In each household, one person was sampled, male or female (switching off each time). For each possible participant, three dial-up attempts were made at various times, after which, if there was no answer, the enumerators underwent a new random sample using the same method. If the interviewee was a parent of a child (age < 15) who lived in the household, s/he was asked to complete the questionnaire on behalf of either the oldest or youngest child living in the house. The survey was conducted in Hebrew, English, Arabic, and Russian.

For each subgroup defined in the strata we had a large representative database, from which the sub-populations that were missing were randomly sampled. After the initial two months of data collection, it was evident that the < 44 age group were not sufficiently represented. Initially, a cellphone database was suggested, but, due to regulatory issues, that list was not available. Therefore, online questionnaires were distributed through an existing representative e-mail database and completed online.

Sample size: The sample size was calculated with a power of 80% for a 95% confidence interval of ± 1.5% deviation, based on the estimated population symptom report rate of 75% and actual medical referral rate of 25%, as defined by White and colleagues [1]. In order to prevent bias due to seasonal illness, stratified samples were implemented throughout an entire year. The samples were constructed using the stratum method, with the criteria for defining the stratum: gender; population, sector, age group, residence and periphery index. Exclusion criteria included those living in sheltered housing, active-duty soldiers and those not fluent in Hebrew, English, Arabic or Russian.

### Independent Variables

- *Population sectors*
  - self-identified immigrants from the Former Soviet Union (FSU) after 1990
  - Long-Term Jewish Residents (LTJR), those born in Israel or immigrated to Israel prior to 1990.
  - Israeli Arabs
  - Others
- *Periphery index* as determined by the Israel Central Bureau of Statistics, was calculated as an aggregate of 2/3 of an accessibility index and 1/3 a measure of proximity to the Tel Aviv district [13].
- *Age* categorized as 15–24, 25–44, 45–64, 65 and older.

### Statistical Methods

Our questionnaire was based on that of Green and colleagues [2], written in Hebrew and modified for the Israeli population, and subsequently translated to Russian and Arabic, using a team of external medical experts. The ecology questionnaire included the following questions: Thinking about the last 30 days, have you experienced any type of medical symptoms, discomforts or injuries in the past month? Did you seek out medical assistance because of this illness? Were you hospitalized? In our survey we also asked if they had experienced any symptoms in the previous two weeks, in order to reduce recall bias as well as a question on perceived risk of developing cardiac or vascular disease: “As far as you know, are you at increased risk of a cardiovascular disease?”. The questionnaire is attached as a Additional file 1.

A pilot study was conducted on 101 participants to validate our questionnaire. Statistical analysis was conducted using the SPSS program (Chicago, IL, v. 23). The correlation between socio-demographic variables (gender, age, sector, and residential area) and the pattern of morbidity and consumption of medical services in the population was examined using a t- test for continuous quantitative variables, and a chi squared categorical variables for a one-variable analysis. Logistic regression was used for multivariate analysis.

The study was approved by the Hadassah Medical center's institutional review board. Informed consent was requested at the beginning of each interview, cooperation and continuation of the conversation was considered informed consent. An option to refrain from answering was given at each question.

## Results

**Fig. 1:**
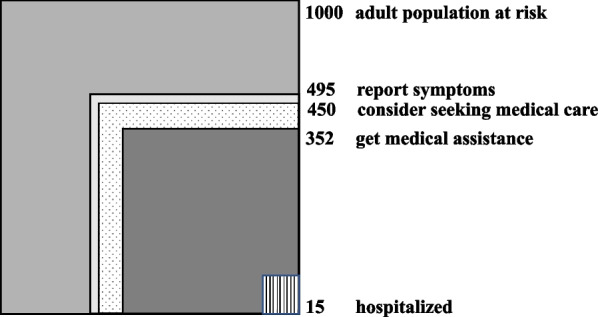
Monthly prevalence of subjective morbidity and medical care consumption in Israel

A total of 1862 interviewees from eleven stratified monthly samples were included. Table 1 shows sociodemographic characteristics of the sample. Overall, for every 1000 adults, 495 reported some type of morbidity, 450 considered seeking out medical care, 352 sought out care, and 15 were hospitalized (Fig. 1).

**Table 1:** Sociodemographic characteristics

		Age group				n (%)
		15–24	25–44	45–64	65 +	
Gender	Male	249	362	169	135	915 (49.1)
	Female	135	316	314	182	947 (50.9)
Sector	Israeli Jews	276	468	345	218	1307 (70.1)
	Immigrants from the FSU	20	97	82	79	278 (14.9)
	Arab Israelis and others^*^	88	113	57	19	277 (14.9)
Education	High school education	321	216	222	149	904 (48.5)
	> 12 years education	29	103	64	45	241 (12.9)
	College degree	35	240	101	51	427 (22.9)
	Higher degree	0	119	96	72	291 (15.6)
Total		384	678	483	317	1862 (100)

*Muslim Arabs, Christian Arabs, members of the Druze community and others

**Fig. 2:**
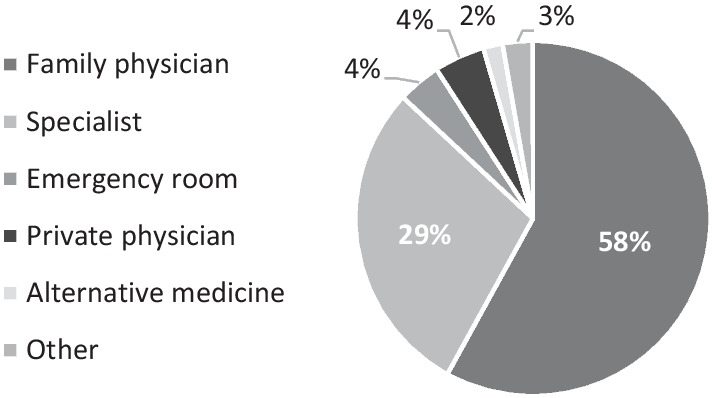
Types of medical assistance consumed

When asked who their preferred source of initial care would be, most of the participants (56.2%) stated their primary care physician. In practice, this was even more evident, as the vast majority (58%) sought out their primary care physician when they chose to pursue medical services (Fig. 2).

**Fig. 3:**
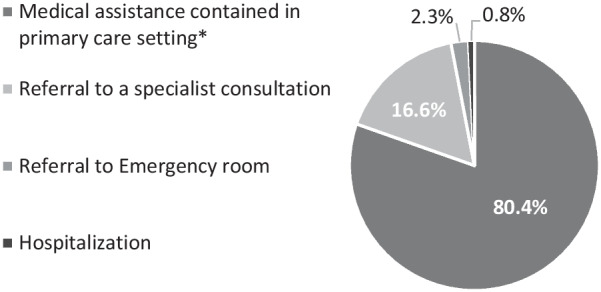
Products of family physician's consultations. ^*^follow-up, prescription, laboratory tests or imaging

Of those participants who went to their family physician, 80% were able to resolve the health issue within the primary care setting, 17% were referred to an additional specialist, and 0.8% were hospitalized (Fig. 3).

**Fig. 4:**
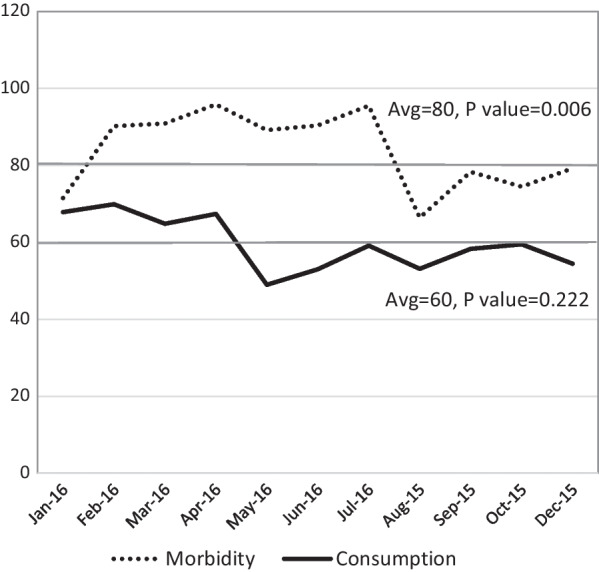
The effect of sampling time (in months) on morbidity and consumption of medical services. Number of participants who reported morbidity or consumption of medical services during a given month appears on the Y-axis

Overall subjective morbidity differed significantly among the population sectors; 53.3% of LTJR reported illness, whereas 50.2% of FSU immigrants and only 31% of Arab Israelis, reported illness in the past month (*p* = 0.000). Gender also affected reporting of subjective morbidity; 53% of women reported illness, as compared with 46% of the men (*p* = 0.015). 38.9% of the women sought out services, as compared with 31.5% of men (*p* = 0.006). Even though subjective morbidity varied throughout the year (*p* = 0.006), actual consumption remained relatively steady (Fig. 4).

Subjective morbidity was statistically associated with increased age, being a Jewish Israeli, having an increased self-perception of cardiovascular morbidity risk and time of year (Table 2). Increase in morbidity reporting was not associated with years of education, smoking status or level of physical activity. Arab Israelis reported lower illness perception, lower subjective morbidity (OR = 0.34, *p* < 0.000), decreased interest in medical assistance and lower consumption of health services than the other groups (OR = 0.48, *p* < 0.000).

**Table 2:** Multivariate logistic regression examining the effect of socio-demographic variables on the prevalence of morbidity, considering seeking care, consumption of medical services and medical services waiver (participants who reported illness but did not get medical assistance)

	Morbidity		Considered care		Consumption		Waiver of medical services	
	OR	*p* value	OR	*p* value	OR	*p* value	OR	*p* value
Gender		0.015		0.006		0.012		0.472
Male	1		1		1		1	
Female	1.28		1.32		1.3		0.90	
Age GROUP		0.002		0.101		0.019		0.123
15–24	1		1		1		1	
25–44	0.92		1.18		1.01		0.90	
45–64	1.3		1.45		1.43		0.68	
65 +	1.56		1.31		1.37		1.09	
Population group		0.000		0.00		0.00		0.814
Jewish Israelis	1		1		1		1	
Immigrants from FSU	0.69		0.91		0.83		0.89	
Arab Israelis and others	0.34		0.48		0.48		0.89	
Education		0.141		0.695		0.74		0.605
High school education	1		1		1		1	
> 12 years education	0.78		0.86		0.85		1.29	
College degree	0.83		0.88		0.94		0.92	
Higher degree	1.1		0.97		1.02		0.99	
Geographic region		0.123		0.624		0.515		0.026
Center	1		1		1		1	
Periphery	1.28		1.05		0.93		1.42	
Illness perception		0.000		0.000		0.000		0.209
None	1		1		1		1	
Yes	1.56		1.85		1.56		0.80	

Of the 837 participants considering seeking out medical care for their symptoms 657 sought out medical assistance (78.5%). Like morbidity, consumption was positively associated with being a woman, older, LTJR and having a higher level of self-perception of cardiovascular morbidity risk. Utilization of health services was not associated with education, time of year, smoking status or physical exercise habits.

When analyzing the population that reported morbidity yet did not seek medical care, only one variable was of statistical significance, geographic location (Table 2). Residents living in the geographic periphery of the country forewent medical services more than those residents living in the center (OR = 1.42, *p* = 0.026). In the south, this concession was even greater (OR 1.52, *p* = 0.029).

## Discussion

**Fig. 5:**
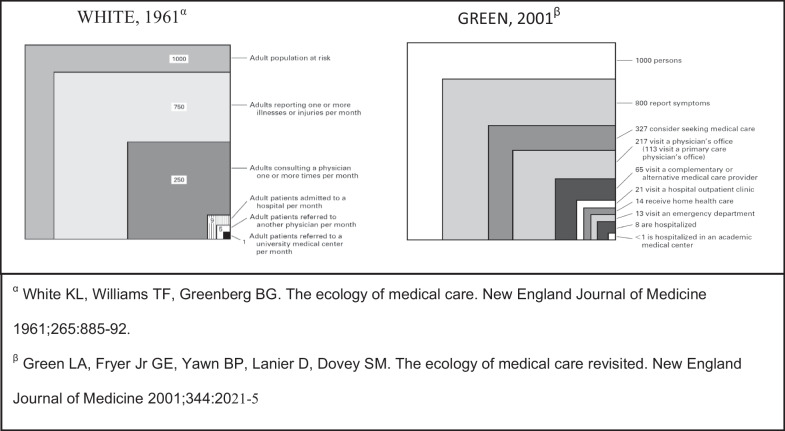
The ecology of medical care models from the past, Presenting the monthly prevalence of subjective morbidity and medical care consumption in the US and England

Reported morbidity in Israel is, comparatively, significantly lower than reported in the literature [3, 4]. Approximately 50% of the Israeli population reported morbidity during the past month as compared with the 70–75% described previously [1, 2], 86% in Japan, and 90.1% in Norway [5]. Actual service consumption, however, was paradoxically higher in our study (352/1000), as seen in Fig. 5, more than Norway (214/1000) [5], the United States, (217/1000) [2], Canada (238/1000) [14], and Japan (307/1000) [3], but not of Hong Kong (440/1000) [4].

It is possible that the perception of morbidity reported in Israel is lower than what is common in the world due to differences in cultural perceptions of illness. In a recent study on self-rated health among Israeli women, immigrants reported lower perceived good health than the veteran Israeli population [15]. Significant differences in healthcare usage have also been seen between other European countries; for example, French citizens were much more likely to visit their physician and receive symptomatic treatment for an upper respiratory infection, than the Dutch [16]. High consumption of medical services indicates good availability of public health system, probably thanks to universal health care coverage. Our findings suggest that Israelis who perceive themselves as sick will usually consider medical assistance and are very likely to receive it. On the other hand, the discrepancy between the peripheral versus central populations seeking out care seems to imply that the health care system in Israel allows for medical assistance when needed but in an unequal fashion. That is, morbidity perception and the rate of those considering pursuing medical assistance were similar between the center of the country and the geographic periphery; however, residents of the periphery forewent seeking out healthcare services significantly more than the rest of the population. In Israel, discrepancies in healthcare usage and barriers to access due to distance and lack of tertiary medical centers have been documented in the past [17, 18]. Interestingly, Arab Israelis reported lower levels of illness perception, morbidity and consumption, despite well documented studies that demonstrate increased morbidity and mortality among the Arab Israeli population and increased risk factors for developing disease [17–23]. The discrepancy found between health perception compared to the high prevalence of morbidity and its complications in Arab society has also been demonstrated in other studies [24, 25]. For example, Arab Israeli women reported relatively high rates of good self-rated health, yet the same population had the highest rate of frequent use of family physician (45%), the same percentage of chronic illness as the Jewish population, and the highest level of depressive symptoms [15]. Self-health perception is related to a variety of factors such as economic well-being, level of education, knowledge, social security and culture. Studies conducted in Israel have shown that all of these have some contribution in creating this gap between Arab and Jewish populations [26–29]. Lack of medical services in the periphery may also widen the gap between known morbidity and consumption of medical services. All these foster frustration, underutilization of medical services and, as a result, poorermedical outcomes. About a decade ago, the Ministry of Health in Israel promoted a strategic plan designed to reduce the gaps between periphery and center and between the various sectors of Israeli society [30]. Funds have been invested in improving access to medical services and bringing advanced technologies to the periphery, promoting health education, publishing information in various languages and implementing an index program to examine the quality of medicine [31]. Change has apparently begun, but there is still a long way to go. Most of the interventions described above have been designed to improve the accessibility of inpatient systems. However, our findings show that 98.5% of medical services are consumed in the community and 80% of all family physician consultations are contained within primary care setting. When a person in the periphery is considering getting medical advice he seeks out his family physician and at this point he is probably facing the most common barrier. Promoting healthy lifestyle such as exercise and smoking prevention, addressing common medical problems and managing chronic diseases such as hypertension and diabetes—are all processes that take place in the community. The pattern of consumption described in this work (the "ecology" of medical care consumption) along with the preferences expressed by the study participants show that investing in primary care may be of great benefit in improving the accessibility of medical services in the periphery and reducing disparities.

### Methodological changes and limitations

Low response rate may create a selection bias. Overall response rate to this survey was 15.6% (calculated by dividing the number of respondents by the number of calls that had to be made until the desired number of respondents was reached, including missed calls and refusals to participate). The response rate was highest among the Arab population (21.8%) and lowest among the immigrant population (12.7%). Young people (under the age of 45) were not available on a landline, which greatly increased the number of calls and affected the overall response rate. For over a decade, it has been evident that landline purchase is declining, especially among those under forty, and being replaced by increased cell phone line usage. This trend is seen in the United States [32, 33], Europe [34], and Israel [35]. Therefore, our decision to modify our sampling methods, midway through the study, and use internet-based questionnaires for this population, was reasonable and necessary given the circumstances. After receiving approval from the Ethics Committee, an online survey was distributed among this stratum using the "I PANEL" database, a large database, with about 100,000 members containing the diverse population strata. 767 questionnaires were distributed in this way to the younger age groups, 402 were completed and returned (52.4%). Due to the difference in reporting styles, self-completed as compared with interview, as well as possible selection bias of the e-mail database, our results may not be representative of the overall 15–44 age group in Israel. In order to reduce memory bias we asked about the incidence of morbidity and consumption of medical services in two stages: first about the last two weeks and then about their incidence during the last month. In order to test the reliability of our results a comparison was made with the 2009 Health Survey of the Central Bureau of Statistics (published in 2013). More than 20,000 participants aged 15 and over took part in this National Survey and their response rate was very high (82%). Still, the results of the surveys are very similar.19.2% of survey participants conducted by the Central Bureau of Statistics reported consulting a physician during the past two weeks compared to 21.3% who sought medical assistance during the same period in our work.

## Conclusions

According to our study, Israeli society reports significantly lower overall morbidity than seen in in other countries, perhaps due to cultural differences in illness perception. On the other hand, consumption of medical services is paradoxically higher. Illness perception and healthcare consumption varies significantly among sub-populations within Israel. However, an Israeli who feels ill will usually consider seeking medical assistance and, in most cases, will seek and receive medical assistance more so than residents in other countries, probably thanks to universal health care.

Israel's universal healthcare system declares that every patient in Israel will receive quality medical assistance, in a timely fashion, within a reasonable distance. The prevalence of morbidity in the central and periphery of the country is similar, yet the percentage of failure to initiate and cessation of medical services is significantly higher in the periphery. These findings indicate that there are still barriers to health service consumption in the State of Israel, affecting specific populations unequally. The contrast between perception of morbidity described in this work and its actual prevalence among the Arab population also requires further interventions of the Ministry of Health and the HMOs. Emphasis should be placed on health education and improving access to primary medical services in the periphery. Promoting healthy lifestyle in different languages, giving preference to public infrastructure that encourages an active lifestyle, enforcing a non-smoking environment and more. Collaborating with local authorities and local influential figures, intellectuals and public activists will help create a change in health perception. A national plan to strengthen primary medicine in the periphery may reduce the gaps described. Improving access by providing grants to family physicians who choose to live or work in the periphery along with improving the work environment in primary care settings (allowing the family physician to perform an adequate and appropriate medical inquiry). Improving employment conditions of primary care providers in the periphery (wages and retirement conditions) may help maintain this solution over time.

## Supplementary Information

**Additional file 1**: The questionnaire used by the researchers, in the English language.

## Data Availability

The datasets generated and/or analyzed during the current study available from the corresponding author on reasonable request.

## Abbreviations

FSU: Former Soviet Union
LTJR: Long-Term Jewish Residents
HMO: Health maintenance organization
