# Supplementary material for: The landscape of medical care consumption in Israel: a nationwide population cross-sectional study
Source: Isr J Health Policy Res. 2022 Nov 10;11:38. doi: 10.1186/s13584-022-00542-9 (PMC9650793; doi:10.1186/s13584-022-00542-9)
Supplement: Supplementary file 1 — Additional file 1: The questionnaire used by the researchers, in the English language. [file 13584_2022_542_MOESM1_ESM.docx]

**The ecology of medical care in Israel: Research questionnaire**

1. **Sociodemographic Information**:
2. **Age**: _______ years
3. **Gender**: M / F
4. **Sector**:
5. Jewish / Arab / Other
6. Born in Israel (or year of immigration is earlier than 1990) / Immigrated from the USSR from the 1990s onwards / Other
7. **Education**: What is the highest school leaving certificate or school degree that you have obtained?
8. Elementary school
9. Secondary school / high school
10. Certificate studies / technician
11. Bachelor's degree (in college or university)
12. Master's degree or higher
13. None
14. Other type
15. **Health insurance**: Which HMO are you a member of? _____________
16. **Living area:**

*Make sure that there is a match between the answers and database's details*

- 1. Urban / rural / kibbutz / other
  2. North / Haifa / Jerusalem / Central (Gush Dan) / South / west bank

1. **Morbidity and Consumption Questionnaire**:
2. **Thinking about the last two weeks, have you suffered from any medical problem: discomfort, pain, injury or illness?** (Yes, no, do not know, refuses to answer***)*** *Any report of a symptom will be a positive answer*
3. **During the last two weeks, have you considered seeking medical assistance for any reason? Even if you did not apply in the end?** (Yes, no, do not know, refuses to answer)
4. **Did you seek medical assistance during this period?** (Yes, no, do not know, refuses to answer)

*(Applying for a certificate is also considered a positive answer)*

1. **Thinking about the last 30 days, have you suffered from any medical problem: discomfort, pain, injury or illness**? (Yes, no, do not know, refuses to answer)

*(Any report of a symptom will be a positive answer)*

1. **During the last 30 days, have you considered seeking medical assistance for any reason? Even if you did not apply in the end?** (Yes, no, do not know, refuses to answer)
2. **Did you seek medical assistance during this period?** (Yes, no, do not know, refuses to answer)

*(Applying for a certificate is also considered a positive answer)*

1. *For those who answered YES to questions 3 or 6:*
2. **What was the treatment you received?** More than one answer can be specified
3. Recommendation for follow-up
4. Prescription for treatment
5. Referral for laboratory tests
6. Referral for imaging examination
7. Referral to a consulting physician
8. Emergency room referral
9. Hospitalization
10. Other__________
11. *For those who answered YES to questions 2 or 5 but NO to questions 3 or 6*: **Why didn't you seek medical assistance?** ________________________________________________
12. **Have you been hospitalized in the last year?** (Yes, no, do not know, refuses to answer)
13. **Who would you prefer to consult in case you were suffering from any medical problem: discomfort, pain, injury or illness?**
14. A Family physician
15. A Pediatrician
16. A specialist in internal medicine
17. A specialist in geriatrics
18. A specialist doctor in another field, depending on the problem I was suffering from
19. Emergency Room
20. Alternative Medicine
21. A nurse
22. Do not know
23. Other: ______________
24. D**o you exercise regularly?** (Yes, no, do not know, refuses to answer)

*For those who answered YES to question 11:*

- - 1. **What kind of activity do you do? ___________________________**
    2. **How many times a week do you exercise? ___________________**
    3. **How long does such exercise last on average? _______________**

*For those who answered NO to question 11:*

- - 1. **Why don't you exercise?** _________________________________

1. **Do you smoke?** (Yes, no, do not know, refuses to answer)
   1. *For those who answered yes to question 12:*

**How many cigarettes do you smoke a day?** _________________

1. **To the best of your knowledge, are you at increased risk for cardiovascular disease?** (Yes, no, do not know, refuses to answer)
2. **Would you be willing to answer another survey on this topic later or participate in a more in-depth interview on the subject?** (Yes, no, do not know, refuses to answer)
3. **Are you a parent of a boy or girl living with you at home?** (Yes, no, do not know, refuses to answer)

*For those who answered YES to question 15:*

- - 1. **Are you ready to answer the questionnaire again, but this time with reference to your child?** (Yes, no, do not know, refuses to answer)
